# Pierre Robin Sequence in a Child With Ectopic Kidney, Polysyndactyly, And Short Stature: A Case Report

**DOI:** 10.7759/cureus.6475

**Published:** 2019-12-27

**Authors:** Anan Abualshamat, Abdulmoein Al-Agha

**Affiliations:** 1 Medicine, King Abdulaziz University Hospital, Jeddah, SAU; 2 Pediatrics, King Abdulaziz University Hospital, Jeddah, SAU

**Keywords:** pierre robin, sequence, syndrome, anomalies, micro-retrognathia, isolated.

## Abstract

Pierre Robin sequence (PRS) is a set of congenital abnormalities commonly comprising small jaw (micrognathia), posteriorly placed tongue (glossoptosis), and airway obstruction; however, other abnormalities may also be associated with PRS. Here we report the case of a 5-year-old girl with short stature, polysyndactyly, and an ectopic kidney who presented with PRS features.

## Introduction

Pierre Robin sequence (PRS) is a combination of congenital anomalies comprising fetal gnatho-glosso-palatoschisis. It was first described by Hilaire in 1822 and included micrognathia, cleft palate, and airway obstruction; this description was followed by that given by Fairbain in 1846 and by Shukowsky in 1911 [1]. Pierre Robin first reported the association between micrognathia and glossoptosis in 1923, which was followed by another case report in 1934 adding cleft palate to the description [2]. In the 1970s, the term “Pierre Robin syndrome” was changed to “Pierre Robin sequence” because the latter term implies a group of clinical findings [3]. The incidence of PRS varies from 1/31206 live births to 1/8060 live births [4-5].Both sex have equal prevalence [6]. PRS can be isolated (iPRS) or part of a genetic syndrome [7]. Severe respiratory distress and failure to thrive, the most common consequences of PRS, are usually secondary to small jaw, particularly when associated with glossoptosis [8-9]. We report a case of a 5-year-old girl with iPRS associated with an ectopic horseshoe kidney, polysyndactyly, and short stature.

## Case presentation

**Figure 1:**
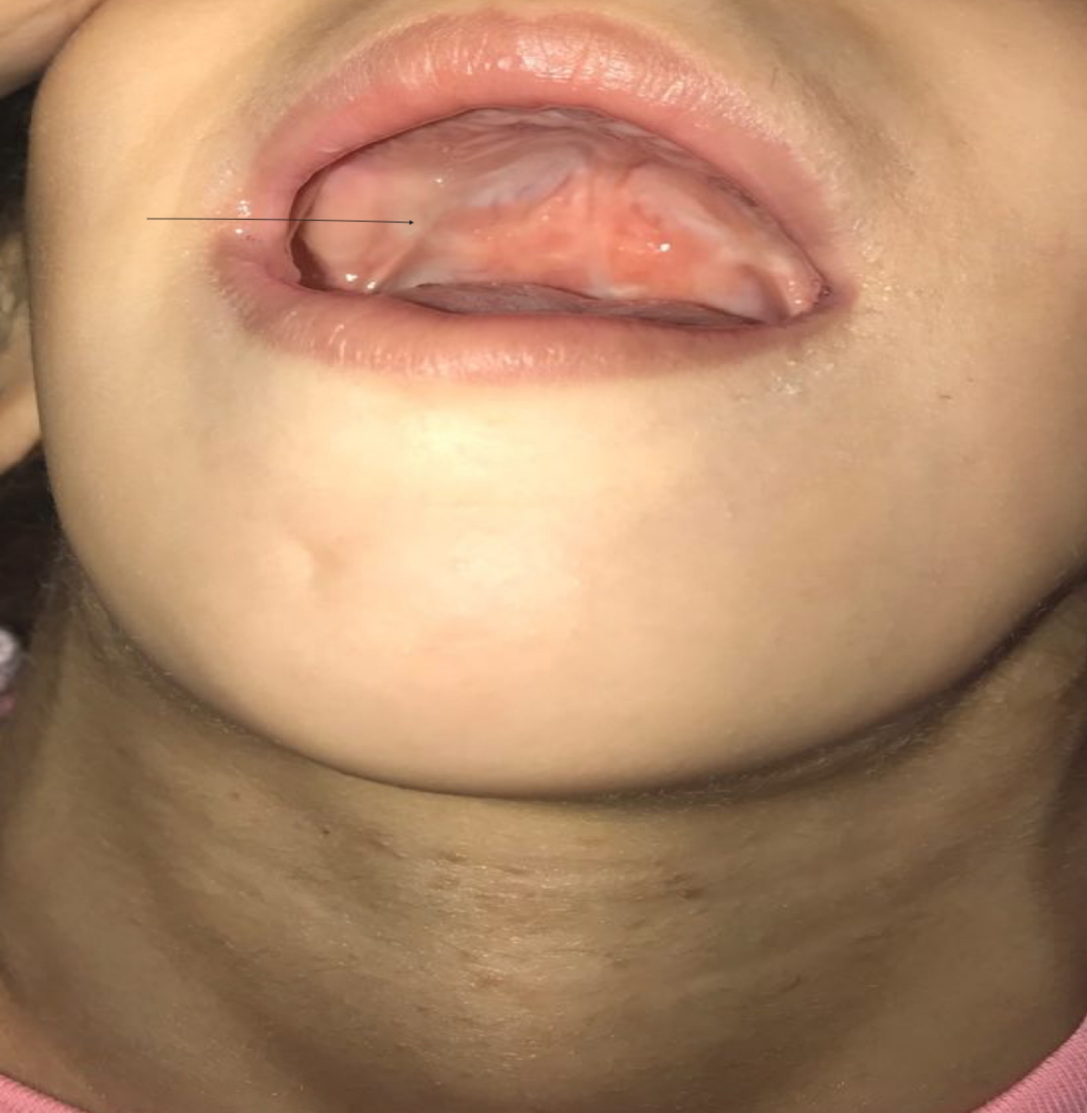
Post cleft palate repair

**Figure 2:**
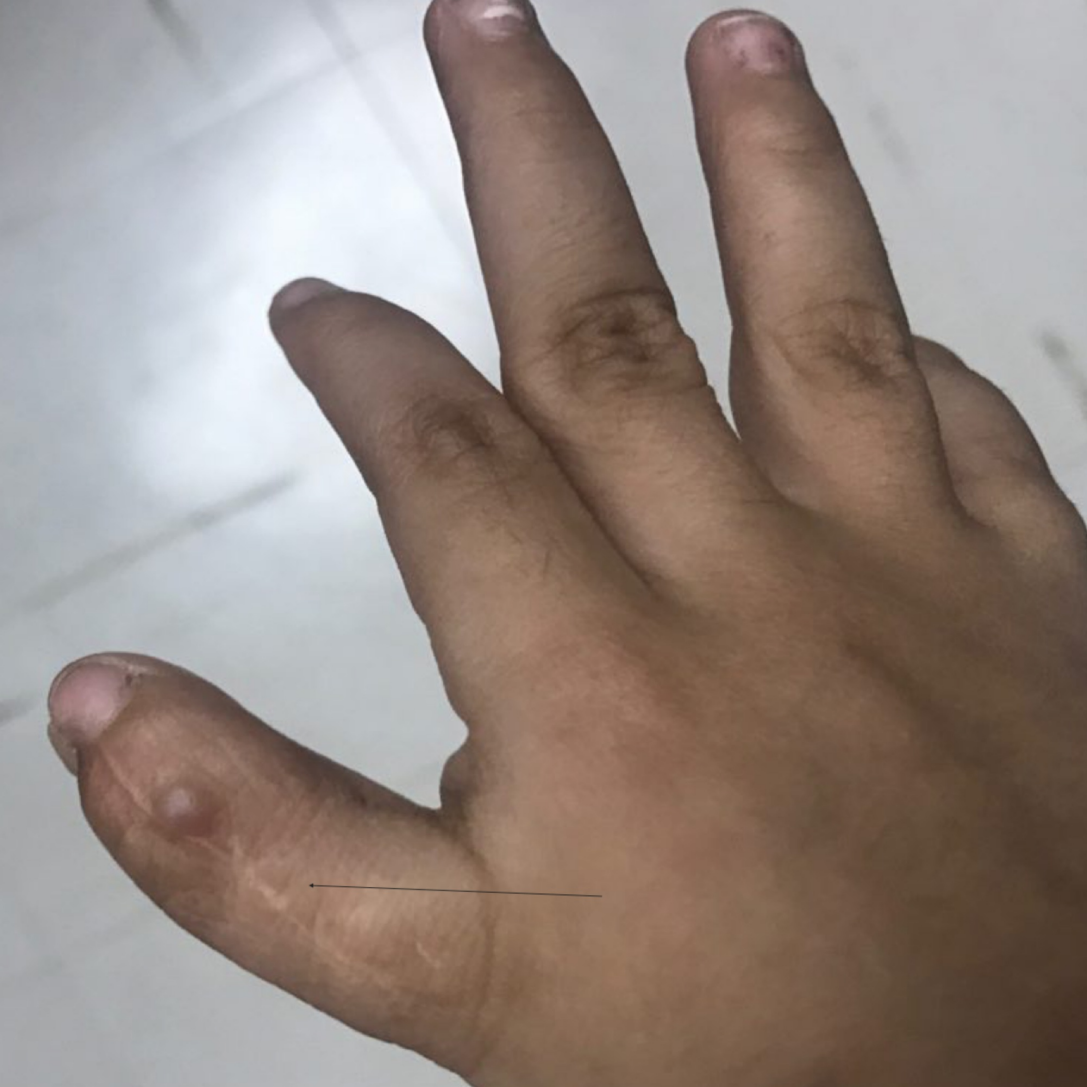
Post reconstruction of the right thumb due to congenital polysyndactyly

**Figure 3:**
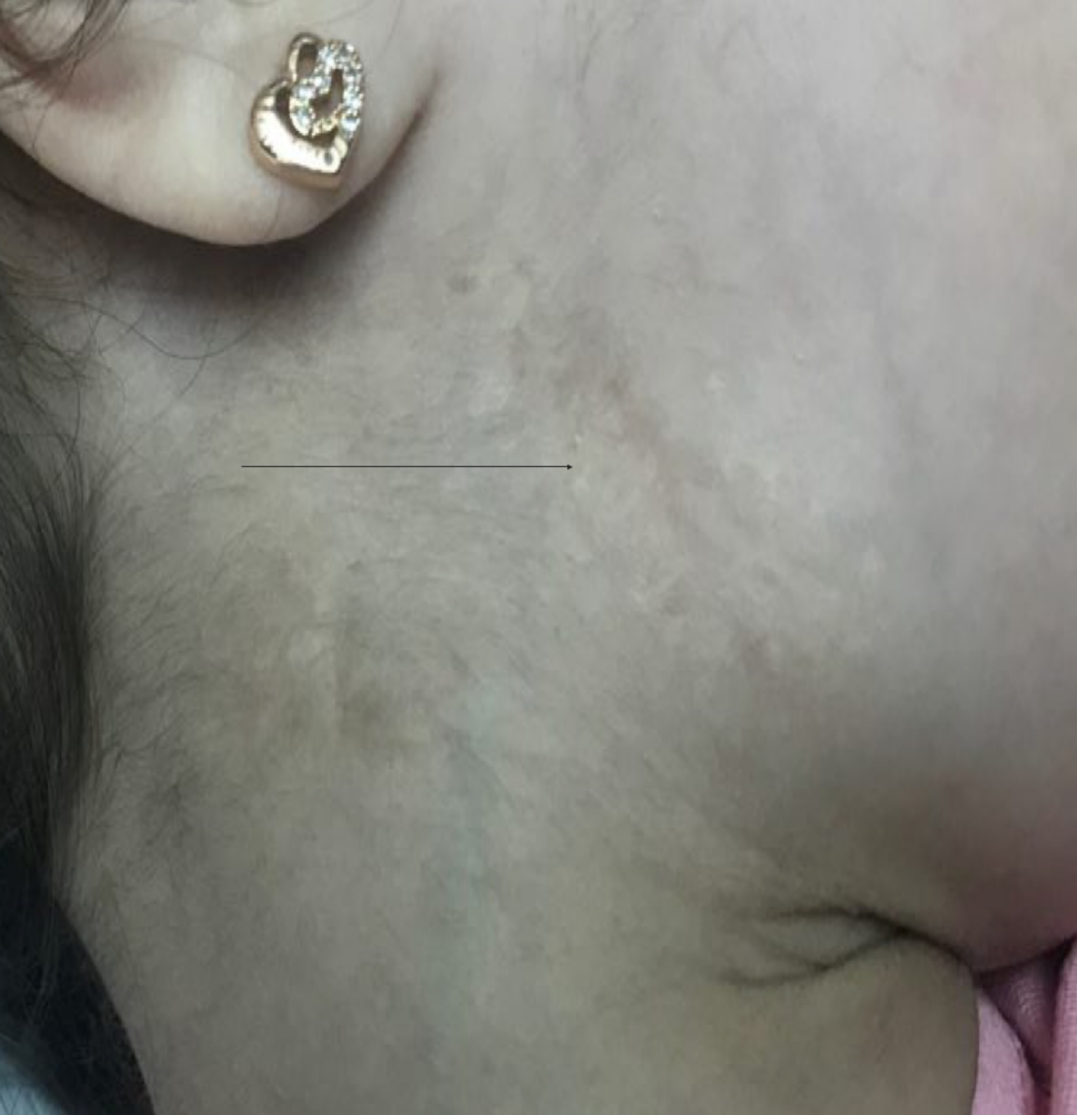
Post bilateral mandibular reconstruction and tongue tie

**Figure 4:**
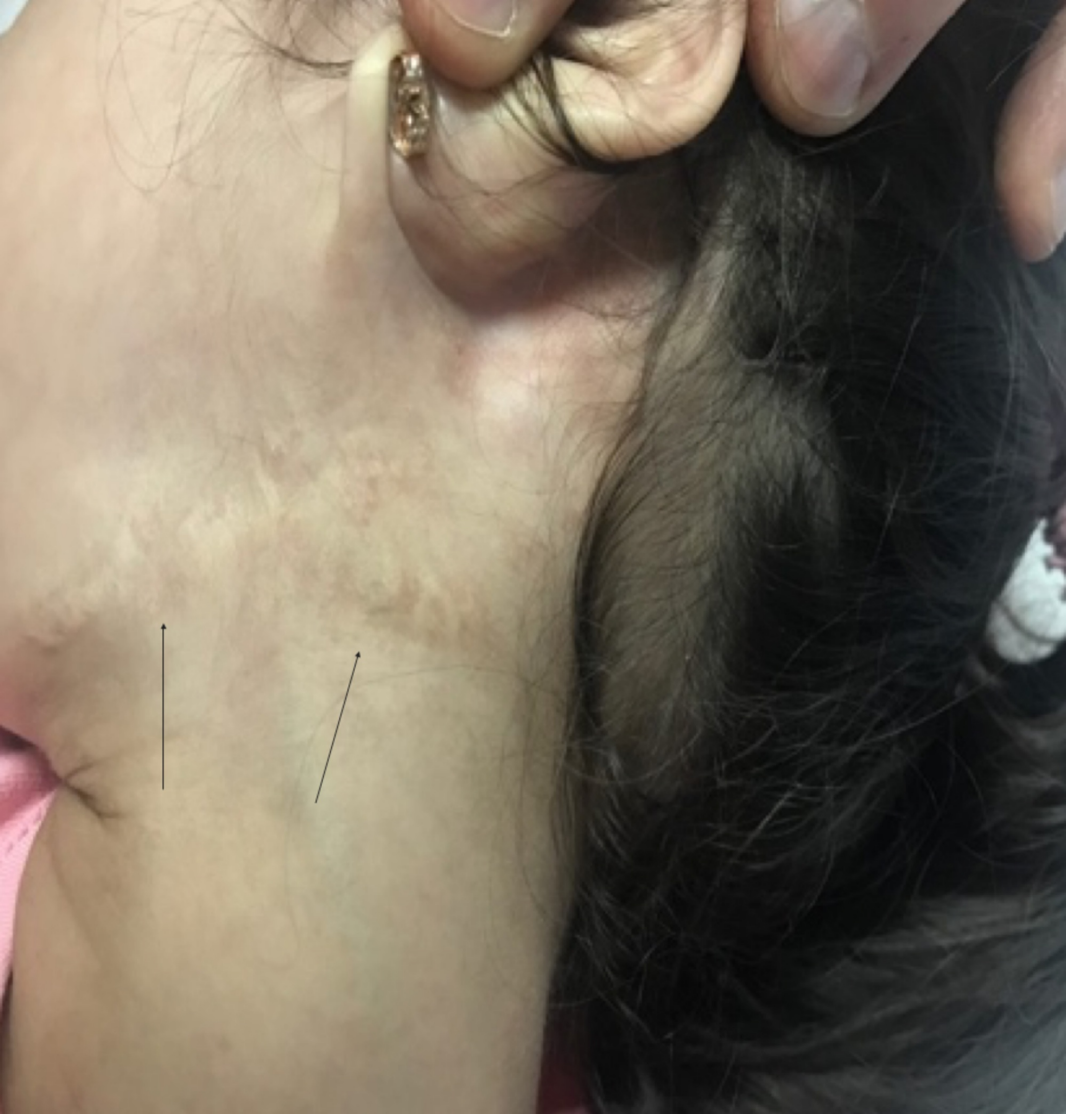
Post bilateral mandibular reconstruction and tongue tie

**Figure 5:**
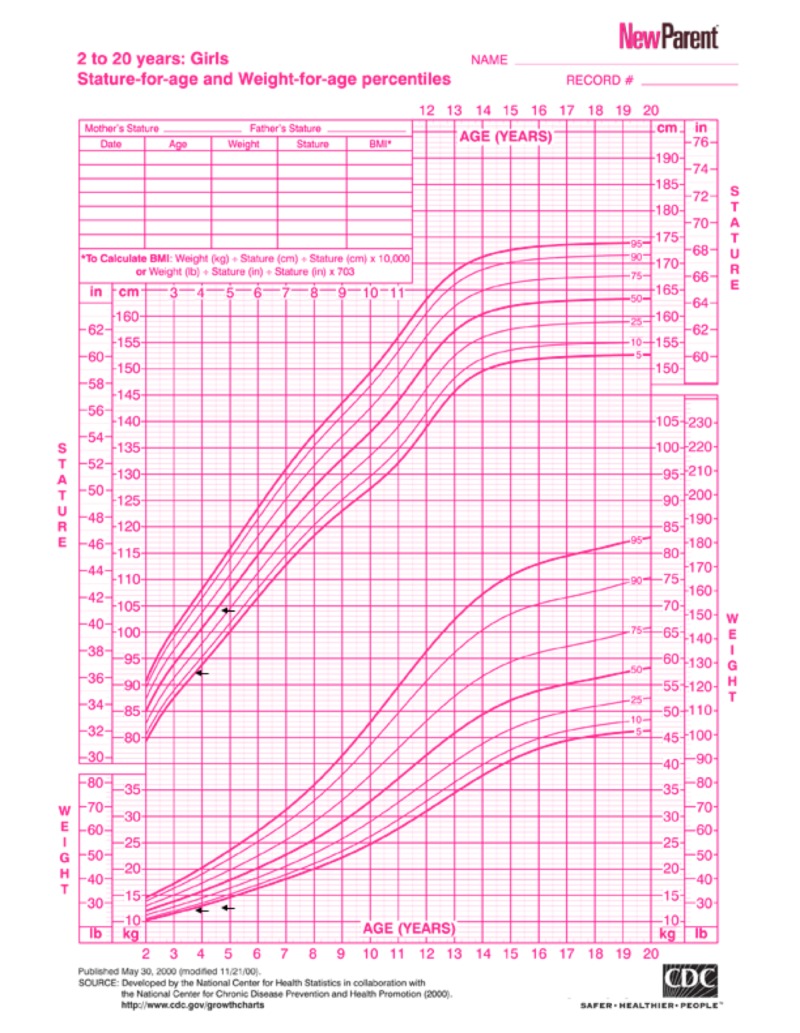
Growth chart showing 92 cm height and 12.20 kg weight, both below the third percentile for the patient’s age and sex, and one-year follow up after growth hormone therapy with height of 104 cm

A 5-year-old Yemeni girl with a known diagnosis of iPRS was delivered at full term via cesarean section. Her parents were first-degree cousins. Her antenatal history was normal; however, her mother had mild preeclampsia, which needed to be treated with antenatal vitamin supplements. She had a low birth weight of 1800 g and a height of 48 cm, which were both below the third percentile for her age and sex. Her parents presented her to the Pediatric Endocrine Clinic with complaints of short stature and poor weight gain, in comparison to her three siblings who were growing normally. Her past medical history revealed symptoms of feeding difficulties, repeated milk regurgitation with shocking attacks, respiratory distress, snoring, and sleep apnea, all due to her initial congenital anomalies of micro-retrognathia and cleft palate. She had no history of glossoptosis as she had congenital tongue tie. Her developmental history revealed dysarthria with delayed language skill without nasal resonance, normal vision, and normal hearing. There was no family history of a similar condition. She had undergone three surgeries previously; the first was for cleft palate repair at the age of one year (Figure 1), the second was reconstruction of the right thumb due to congenital polysyndactyly at the age of three years (Figure 2), and the third was reconstruction of the mandible and tongue tie at the age of three years (Figures 3-4). On examination, her height was 92 cm and weight was 12.20 kg, which were both below the third percentile for her age and sex (Figure 5). She had no dysmorphic features apart from mild micro-retrognathia, post-bilateral mandible reconstruction (distraction osteogenesis) neck scars, and a reconstructed right thumb scar. Other systemic examination results were normal. Her laboratory investigation showed normal complete blood count, normal thyroid as well as liver and renal function test results, and normal insulin-like growth factor levels.

Imaging modalities were used to rule out other associated abnormalities; her bone mineral density was 2.4 standard deviations below the expected normal range for her age. Renal ultrasound showed an ectopic horseshoe kidney at the superior midline and a renogram scintigraph demonstrated adequate flow to the ectopic kidney, with no evidence of obstruction. Results of other systematic investigations were normal. Growth hormone therapy was started because she was small for gestational age and owing to failure to catch up in terms of growth in the first three years, as indicated by the Food and Drug American Association. One year after receiving growth hormone therapy, her height improved to 104 cm (Figure 5).

## Discussion

Here we report a case of a 5-year-old girl with short stature, polysyndactyly, and an ectopic kidney who presented with PRS features.

The name PRS was derived from one known or presumed single anomaly or mechanical factor, but it can cause multiple anomalies [10]. PRS presents as a group of variable physical findings that are different causally, pathologically, and phenotypically from patient to patient [11-12]. PRS begins with an undersized jaw (micrognathia) or an abnormal posterior position (retrognathia) of the mandible that restricts the space for the developing tongue. As a result, the tongue is forced to be placed toward the back or be in an elevated position in the oropharynx (glossoptosis), leading to delayed elevation/fusion of the palatal shelves (cleft palate) and respiratory distress. The mandibular anomaly can be the result of an associated congenital anomaly in 60% of the cases or may result from an abnormal foetal position, uterine anomalies, oligohydramnios, or foetal crowding in iPRS in 40% of the patients [13-14]. The most common syndrome that may be associated with PRS is Stickler syndrome [11]. The initial presentation of PRS includes 100% of retrognathia, 76% glossoptosis, 85% cleft palate, 92% feeding/swallowing difficulties, and 89% breathing difficulties [15], all of which were present in our case, except glossoptosis. Other unusual findings consist of macroglossia and ankyloglossia in 10%-15% of cases, auricular malformations and otitis media in 75%-80% of cases, nasal deformities (especially anomalies of the nasal root) in 33% of cases, and laryngomalacia and gastroesophageal reflux with esophagitis in 10%-15% of cases [16]. Systematic anomalies are present in 10%-80% of the cases, which include 10%-30% of cases with ocular, 5%-58% with cardiovascular, 70%-80% with musculoskeletal, 50% with neurological, and 10%-25% with genitourinary anomalies [14]. The following presentations were found in our case: micro-retrognathia, cleft palate, polysyndactyly, short stature, poor weight gain, and ectopic horseshoe kidney without any other systemic anomalies. An ectopic horseshoe kidney is a rare presentation in PRS cases as determined based on a review of the literature, which revealed that hydronephrosis is the only renal abnormality associated with PRS accounting for 15% of cases [16]. The renal abnormality could also be explained by a family history of a single functioning kidney in a grandfather, which could have been inherited or may be attributable to a rare association. The short stature in our patient could also either be attributable to a rare association or her being small for gestational age status [17].

## Conclusions

Infants and children with isolated or syndromic type of PRS should be evaluated for the presence of renal anomalies during early assessment and follow-up to prevent further kidney injury. Patient stature evaluation should also be considered for any abnormalities to administer growth hormone therapy when indicated.

## References

[1] St. Hilaire H, Buchbinder D (2000). Maxillofacial pathology and management of Pierre Robin sequence. Otolaryngol Clin North Am.

[2] Robin P (1934). Glossoptosis due to atresia and hypotrophy of the mandible. Am J Dis Child.

[3] Sadewitz VL (1992). Robin sequence: changes in thinking leading to changes in patient care. Cleft Palate Craniofac J.

[4] Vatlach S, Maas C, Poets CF (2014). Birth prevalence and initial treatment of Robin sequence in Germany: a prospective epidemiologic study. Orphanet J Rare Dis.

[5] Scott AR, Mader NS (2014). Regional variations in the presentation and surgical management of Pierre Robin sequence. Laryngoscope.

[6] Andreas P, Andersen M (2004). Pierre Robin sequence in Denmark: a retrospective population-based epidemiological study. Cleft Palate Craniofac J.

[7] Cicchetti R, Cascone P, Caresta E (2012). Mandibular distraction osteogenesis for neonates with Pierre Robin sequence and airway obstruction. J Matern Fetal Neonatal Med.

[8] Linz A, Bacher M, Urschitz MS, Buchenau W, Arand J, Poets CF (2011). Diagnostik und therapie der Pierre-Robin-sequenz [Article in English, German]. Monatsschr Kinderheilkd.

[9] Sher AE (1992). Mechanisms of airway obstruction in Robin sequence: implications for treatment. Cleft Palate Craniofac J.

[10] Spranger J, Benirschke K, Hall JG (1982). Errors in morphogenesis: concepts and terms. J Pediatr.

[11] Cohen MM Jr (1999). Robin sequence and complexes: causal heterogeneity and pathogenetic/phenotypic variability. Am J Med Genet.

[12] Pruzanski S (1969). Not all dwarfed mandibles are alike. Birth Defects Res.

[13] Izumi K, Konczal LL, Mitchell AL, Jones MC (2012). Underlying genetic diagnosis of Pierre Robin sequence: retrospective chart review at two children‘s hospitals and a systematic literature review. J Pediatr.

[14] Chiriac A, Dawson A, Krapp M, Axt-Fliedner R (2008). Pierre-Robin syndrome: a case report. Arch Gynecol Obstet.

[15] Vatlach S, Maas C, Poets CF (2014). Birth prevalence and initial treatment of Robin sequence in Germany: a prospective epidemiologic study. Orphanet J Rare Dis.

[16] www.emedicine.medscape.com (2019)Accessed on October 30, 2019 2019 (2019). Pierre Robin syndrome. https://emedicine.medscape.com/article/844143-overview.

[17] Williams AJ, Williams MA, Walker CA, Bush PG (1981). The Robin anomalad (Pierre Robin syndrome)-a follow up study. Arch Dis Child.

